# Betulinic Acid Derivative BA5, Attenuates Inflammation and Fibrosis in Experimental Chronic Chagas Disease Cardiomyopathy by Inducing IL-10 and M2 Polarization

**DOI:** 10.3389/fimmu.2019.01257

**Published:** 2019-06-11

**Authors:** Cássio Santana Meira, Emanuelle De Souza Santos, Renan Fernandes do Espírito Santo, Juliana Fraga Vasconcelos, Iasmim Diniz Orge, Carolina Kymie Vasques Nonaka, Breno Cardim Barreto, Alex Cleber Improta Caria, Daniela Nascimento Silva, José Maria Barbosa-Filho, Simone Garcia Macambira, Diogo Rodrigo Magalhães Moreira, Milena Botelho Pereira Soares

**Affiliations:** ^1^FIOCRUZ, Gonçalo Moniz Institute, Salvador, Brazil; ^2^Science and Health Institute, Federal University of Bahia (UFBA), Salvador, Brazil; ^3^Center for Biotechnology and Cell Therapy, Hospital São Rafael, Salvador, Brazil; ^4^Laboratory of Pharmaceutical Technology, Federal University of Paraíba (UFPB), João Pessoa, Brazil

**Keywords:** *Trypanosoma cruzi*, betulinic acid derivative, immunomodulation, chagas disease, cardiomyopathy

## Abstract

Chronic Chagas disease cardiomyopathy (CCC) is a major cause of heart disease in Latin America and treatment for this condition is unsatisfactory. Here we investigated the effects of BA5, an amide semi-synthetic derivative betulinic acid, in a model of CCC. Mice chronically infected with *T. cruzi* were treated orally with BA5 (10 or 1 mg/Kg), three times per week, for 2 months. BA5 treatment decreased inflammation and fibrosis in heart sections but did not improve exercise capacity or ameliorate cardiac electric disturbances in infected mice. Serum concentrations of TNF-α, IFN-γ, and IL-1β, as well as cardiac gene expression of pro-inflammatory mediators, were reduced after BA5 treatment. In contrast, a significant increase in the anti-inflammatory cytokine IL-10 concentration was observed in BA5-treated mice in both tested doses compared to vehicle-treated mice. Moreover, polarization to anti-inflammatory/M2 macrophage phenotype was evidenced by a decrease in the expression of NOS2 and proinflammatory cytokines and the increase in M2 markers, such as Arg1 and CHI3 in mice treated with BA5. In conclusion, BA5 had a potent anti-inflammatory activity on a model of parasite-driven heart disease related to IL-10 production and a switch from M1 to M2 subset of macrophages.

## Introduction

Chagas disease, caused by the flagellate protozoan *Trypanosoma cruzi*, affects 7 million people worldwide (1). Endemic in Latin American countries, it is increasingly found in non-endemic countries due to intense flow of migration, representing a major public health problem (2). The acute phase of Chagas disease is characterized by the presence of *T. cruzi* parasites in the bloodstream, which trigger an intense inflammatory response in several tissues, especially in the cardiac tissue (3, 4). The majority of *T. cruzi*-infected patients survive in the acute phase and develop a chronic asymptomatic infection (5). Nonetheless, after a variable period of time (10–30 years after the onset infection), about 30% of chronically-infected patients become symptomatic (6).

Chronic Chagasic cardiomyopathy (CCC) is the most common symptomatic form of Chagas disease and may evolve with several manifestations, including heart failure, arrhythmias and thromboembolism (7). CCC is a major cause of heart disease and a cardiovascular-related death in Latin America, and causes a significant economic and social burden in affected countries (8).

Antiparasitic treatment is based on the use of two drugs, benznidazole, and nifurtimox. Although benznidazole has good cure rates when administered during the acute phase, its prolonged use is related to severe side effects, and has limited efficacy during the chronic phase (9, 10). New candidate drugs are being tested, including the inhibitor of ergosterol posaconazole, which failed in promote cure in patients in the chronic stage of disease (11). This scenario makes the search for new drugs with a better profile of safety and efficiency an important issue for Chagas disease.

In addition to eliminating the parasites, an ideal treatment for chronic stage must be able modulate the inflammatory process responsible to the establishment of myocarditis, causing loss of cardiomyocytes and fibrosis deposition, ultimately leading to heart failure, arrhythmias and death of Chagasic patients (12). In this context, the compound BA5, an amide semi-synthetic betulinic acid derivative, is an attractive option. Recently, our group reported the antiparasitic activity against *T. cruzi* trypomastigotes and intracellular amastigotes, with potency similar to benznidazole (13). We also found a potent immunomodulatory activity of BA5 *in vitro*, decreasing the production of crucial inflammatory mediators, including nitric oxide (NO), tumor necrosis factor alpha (TNF-α) and inhibiting the activation of nuclear factor-κB (NF-κB), a transcription factor that regulates the expression of several pro-inflammatory genes (14). Moreover, BA5 conferred protection against a lethal dose of LPS and decreased edema in a delayed type hypersensitivity model (14).

In the present study we tested the therapeutic effects of BA5, a compound with combined antiparasitic and immunomodulatory activities, in a mouse model of chronic *T. cruzi* infection, which reproduces key features of CCC.

## Materials and Methods

### Drugs

Semi-synthetic compound BA5 (95% purity by HPLC) was prepared from betulinic acid, as previously described (13). Benznidazole (LAFEPE, Recife, Brazil) was used as a positive control.

### Animals

C57BL/6 mice (4 weeks old) were bred and maintained in the animal facility of the Center for Biotechnology and Cell Therapy, Hospital São Rafael (Salvador, Bahia, Brazil), and provided with rodent diet and water *ad libitum*.

### *Trypanosoma cruzi* Infection and BA5 Treatment

**Figure 1:**
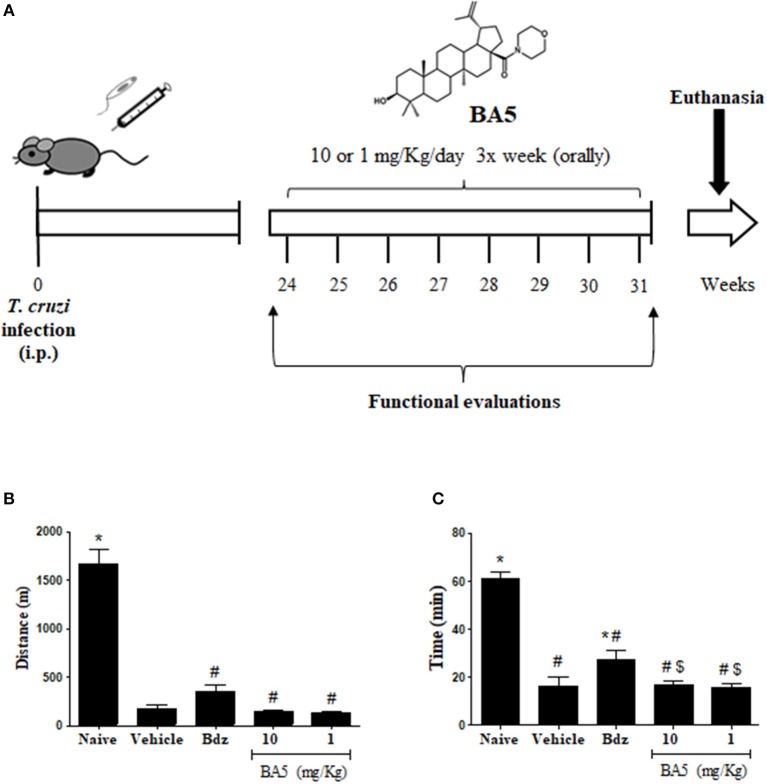
Experimental design and exercise capacity of mice from the different experimental groups. **(A)** C57BL/6 mice were infected with 10^3^ Colombian strain *T. cruzi* trypomastigotes and treated during the chronic phase (6 months after infection) with 10 or 1 mg/kg of BA5 or benznidazole (100 mg/Kg), as indicated. **(B,C)** Distance run and time of exercise on a motorized treadmill. Values represent the means ± S.E.M. of 5 mice (naïve) or 10 mice (infected) per group. *P < 0.05 compared to vehicle-treated mice; ^#^*P* < 0.05 compared to naive group. ^*$*^*P* < 0.05 compared to benznidazole-treated group.

Trypomastigotes of the myotropic Colombian *T. cruzi* strain were obtained from culture supernatants of infected LLC-MK2 cells. Infection was performed by intraperitoneal inoculation of 10^3^ parasites in 100 μL of saline solution and parasitemia was monitored during infection, using standard protocols (15). After 6 months of infection, mice were divided into groups of 10 and received treatments, as follows: 10 or 1 mg/kg of BA5, 100 mg/Kg benznidazole or vehicle (10% DMSO in saline), given orally three times per week for 2 months (Figure 1A). A naive group (*n* = 5) was also included as a control. Mice were euthanized 1 week after therapy, under anesthesia with 5% ketamine and 2% xylazine (Vetanarcol® and Sedomin®, respectively; Konig, Avellaneda, Argentina).

### Exercise Capacity Analysis and Electrocardiography

A motor-driven treadmill chamber for one animal (LE 8700; Panlab, Barcelona, Spain) was used to exercise the animals. The speed of the treadmill and the intensity of the shock (mA) were controlled by a potentiometer (LE 8700 treadmill control; Panlab). Total running distance and time of exercise were recorded. Electrocardiography was performed using the Bio Amp PowerLab System (PowerLab 2/20; ADInstruments, Castle Hill, NSW, Australia), recording the bipolar lead I. All data were acquired for computer analysis using Chart 5 for Windows (PowerLab). The EKG analysis included heart rate, PR interval, P wave duration, QT interval, QTc, and arrhythmias. The QTc was calculated as the ratio of QT interval by square roots of RR interval (Bazett's formula) (16).

### Morphometric Analysis

The hearts of all mice were removed and half of each heart was fixed in 10% buffered formalin. Sections of paraffin-embedded tissue were stained by the standard hematoxylin-eosin and Sirius red staining methods for evaluation of inflammation and fibrosis, respectively, by optical microscopy. Images were digitized using a color digital video camera (CoolSnap, Montreal, Canada) adapted to a BX41 microscope (Olympus, Tokyo, Japan). Morphometric analyses were performed using the software Image Pro Plus v.7.0 (Media Cybernetics, San Diego, CA). The inflammatory cells were counted in 10 fields (400x magnification) per heart. The percentage of fibrosis was determined using Sirius red-stained heart sections and the Image Pro Plus v.7.0 Software to integrate the areas, 10 random fields per animal were captured using a 200x magnification.

### Real Time Reverse Transcription Polymerase Chain Reaction (qRT-PCR)

RNA was extracted of the heart samples using TRIzol (Invitrogen, Molecular Probes, Eugene, OR). cDNA was synthetized using High Capacity cDNA Reverse Transcription Kit (Applied Biosystems). The qPCR was prepared with TaqMan® Universal PCR Master Mix (Applied Biosystems). qRT-PCR assays were performed to detect the expression levels of, *Lgals3* (Mm_00802901_m1)*, Tnf* (Mm_00443258_m1), *IFN-*γ (Mm_00801778_m1), *Il10* (Mm_00439616_m1), *Tbet* (Mm_00450960_m1), *Foxp3* (Mm_00475162_m1), *Gata-3* (Mm_00484683_m1), *TGF-*β (Mm_00441724_m1), *Nos2* (Mm_01309898_m1), C*hi3l3* (Mm_00657889_m1), *Il1*β (Mm_0044228_m1), and *Arg1* (Mm_00475988_m1). All reactions were run in triplicate on an ABI 7500 Real Time PCR System (Applied Biosystems) under standard thermal cycling conditions. A non-template control (NTC) and non-reverse transcription controls (No-RT) were also included. The samples were normalized with *Gpdh* (mm99999915_g1). The threshold cycle (2-ΔΔCt) method of comparative PCR was used to analyse the results (17).

### Parasitemia and Quantification of Parasite Load

Blood parasitemia of infected mice was monitored weekly in the period of treatment by counting the number of motile parasites in 5 μL of fresh blood sample drawn from the lateral tail veins, as recommended by standard protocol (15).

To evaluate the tissue parasitism, *T. cruzi* DNA was quantified in spleen samples by qPCR analysis. For DNA extraction, spleen pieces were submitted to DNA extraction using the Mini Spin plus Kit (Biopur, Cambridge, USA), as recommended by the manufacturer. Spleen samples was submitted to DNA extraction, and the DNA amount and purity (260/280 nm) were analyzed by Nanodrop 2000 spectrophotometry (Thermo Fisher Scientific, Waltham, MA, USA). Oligonucleotides were designed based on the literature and the amounts used per reaction 0.4 μM of both primers foward 5′-GTTCACACACTGGACACCAA-3′ and reverse 5′-TCGAAAACGATCAGCCGAST-3′ and a 0.2 μM of the probe SatDNA specific probe, 5′-/56-FAM/AATTCCTCC/ZEN/AAGCAGCGGATA/3IABkFQ/-3′, (18) and the quantification of parasite load was performed as described previously (19). To calculate the number of parasites per milligram of tissue, each plate contained a 7-log standard curve of DNA extracted from trypomastigotes of the Colombian *T. cruzi* strain (ranging from 10^−1^ to 10^6^) in triplicate. Data were analyzed using 7500 software 2.0.1 (Applied Biosystems).

### Assessment of Cytokine Production

Blood samples was collected via the brachial plexus, centrifuged (1200 g, 10 min, 4°C) and the serum supernatant transferred to microcentrifuge tube and stored at −80 °C until subsequent analysis. Serum samples from the *in vivo* study were used for TNF-α, IFN-γ, IL-1β, and IL-10 determination. Quantification of cytokines was performed by ELISA, using specific antibody kits (R&D Systems, Minneapolis, MN), according to manufacturer's instructions.

### Statistical Analyses

All continuous variables are presented as means ± SEM. Data were analyzed using one-way ANOVA, followed by Newman-Keuls multiple-comparison test with Prism 5.01 (GraphPad Software, San Diego, CA). Differences were considered significant when the values of *P* were < 0.05.

## Results

### BA5 Decreases Cardiac Inflammation and Fibrosis in *T. cruzi*-infected Mice

To investigate the effects of BA5 in chronic Chagas disease, we treated *T. cruzi*-infected mice with BA5 (1 or 10 mg/Kg) benznidazole (100 mg/Kg) or vehicle (Figure 1A). First, we performed functional evaluation by ergometry and ECG before and after treatment. The exercise capacity of mice from both BA5-treated groups showed no improvements compared to saline treated chagasic controls (Figures 1B,C). Treatment with benznidazole caused only a small improvement in time run (Figure 1B). The evaluation of conduction disturbances did not show significant differences between the groups infected with *T. cruzi* before and after treatment, which presented alterations such as atrioventricular block, atrioventricular dissociation and tachycardia (Table 1).

**Table 1:** ECG analysis in naive and *T. cruzi*-infected mice.

**ECG findings**	**Naïve**	**Vehicle**	**Bdz** **100 mg/Kg**	**BA5** **10 mg/Kg**	**BA5** **1 mg/Kg**
	**(*n* = 5)**	**(*n* = 10)**	**(*n* = 8)**	**(*n* = 10)**	**(*n* = 10)**
No alterations	5/5	0/10	0/8	0/10	0/10
AVB	0/5	2/10	0/8	6/10	3/10
AVD	0/5	4/10	6/8	10/10	10/10
EXTS	0/5	0/10	1/8	0/10	0/10
IVCD	0/5	2/10	1/8	0/10	0/10
JR	0/5	0/10	1/8	0/10	0/10
PVT	0/5	2/10	0/8	0/10	1/10
SVT	0/5	2/10	0/8	0/10	0/10

*AVB, atrioventricular block; AVD, atrioventricular dissociation; Bdz, benznidazole; ECG, electrocardiography EXTS, extrassistole; IVCD, intraventricular conduction delay; JR, junctional rhythm; PVT, polymorphic ventricular tachycardia; SVT, supraventricular tachycardia*.

**Figure 2:**
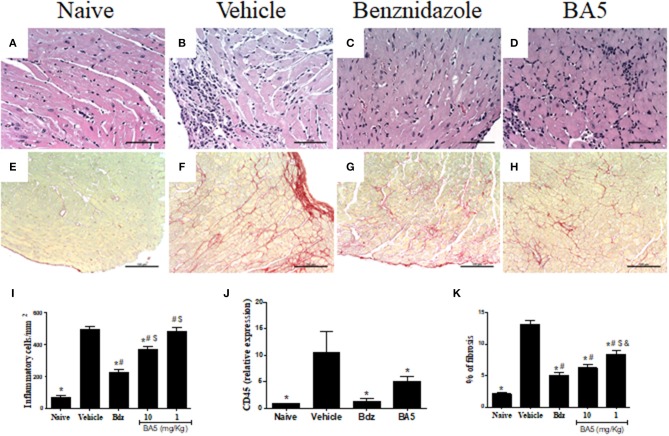
Reduction of inflammation and fibrosis by BA5 in cardiac tissue of chagasic mice. **(A,E)** heart sections of naive group. **(B,F)** heart sections of mice infected with *T. cruzi* and treated with vehicle. **(C,G)** heart sections of infected mice treated with 10 mg/Kg of BA5. **(D,H)** heart sections of mice infected and treated with 100 mg/Kg of benznidazole. **(A–D)** staining with hematoxylin & eosin. **(E–H)** staining with picrosirius red. **(I)** Inflammatory cells were quantified in heart sections of naive mice, vehicle-treated mice, BA5-treated mice or benznidazole-treated mice and integrated by area. **(J)** The expression of CD45 was evaluated by real-time qRT-PCR using cDNA samples prepared from mRNA extracted from hearts of experimental groups. **(K)** Fibrotic area is represented by percentage of collagen deposition in heart sections. Values represent the means ± S.E.M. of 5 mice (naïve) or 10 mice (infected) per group. *P< 0.05 compared to vehicle-treated mice; ^#^*P* < 0.05 compared to naive group. ^*$*^*P* < 0.05 compared to benznidazole-treated group. ^&^*P* < 0.05 compared to mice treated with 10 mg/Kg for BA5.

We next evaluated the effects of BA5 administration in the cardiac tissue, by analyzing heart sections stained with hematoxylin and eosin and Sirius red for quantification of inflammation and fibrosis, respectively. A multifocal inflammatory response, mainly composed of mononuclear cells, was found in vehicle-treated infected mice compared to naïve controls (Figures 2A,B,I). BA5 administration at the highest dose (10 mg/Kg), but not at 1 mg/Kg, decreased the number of inflammatory cells (Figures 2D,I). Treatment with the standard drug, benznidazole, promoted a more pronounced reduction in inflammation (Figures 2C,I). In addition, the gene expression of CD45, a pan leukocyte marker, was increased in *T. cruzi* infected mice treated with vehicle, but not in BA5 (10 mg/Kg) and benznidazole-treated mice (Figure 2J).

Similarly, fibrosis deposition was increased in chronic chagasic hearts compared to naïve controls (Figures 2E,F,K). A significant reduction of fibrosis was found in mice treated with BA5 at 10 and 1 mg/Kg, although the highest dose promoted a more pronounced effect (Figures 2H,K). Benznidazole also reduced cardiac fibrosis compared to vehicle-treated mice, as revealed by Sirius red staining (Figures 2G,K).

### BA5 Modulates the Production of Inflammatory Mediators in Chagasic Mice

**Figure 3:**
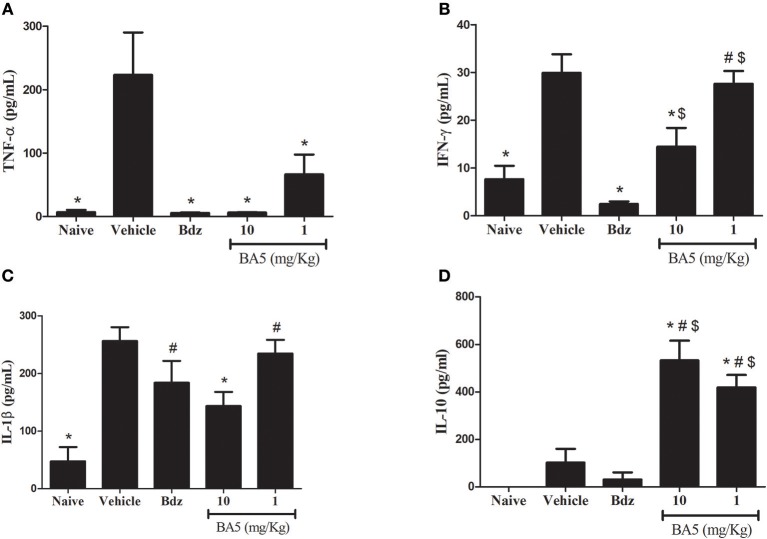
Modulation of systemic cytokine production in chronic chagasic mice treated with BA5 or benznidazole. Effects of BA5 (10 or 1 mg/Kg) or benznidazole (Bdz; 100 mg/Kg) in serum concentrations of TNF-α **(A)**, IFN-γ **(B)**, IL-1β **(C)** and IL-10 **(D)**. Values represent the means ± S.E.M. of 5 mice (naïve) or 10 mice (infected) per group. *P< 0.05 compared to vehicle-treated mice; ^#^*P* < 0.05 compared to naive group. ^*$*^*P* < 0.05 compared to benznidazole-treated group.

The inflammatory response in CCC has been associated with an elevated production of proinflammatory cytokines, such as TNF-α and IFN-γ (20). Thus, we evaluated the effects of BA5 in cytokine production in the sera and in the hearts of chagasic mice. *T. cruzi* chronic infection caused a significant increase in serum concentrations of proinflammatory cytokines TNF-α, IFN-γ, and IL-1β (Figures 3A–C). BA5 promoted a marked reduction in the production of TNF-α in both doses tested (10 and 1 mg/Kg), and a significant decrease in the production of IFN-γ and IL-1β in the highest dose (Figures 3A–C). Benznidazole administration significantly inhibited serum TNF-α and IFN-γ, but not IL-1β. In contrast, a significant increase in the concentration of IL-10, an antiinflammatory cytokine, was observed in BA5-treated mice in both doses tested, compared to vehicle-treated mice. Benznidazole treatment did not induce an increase in IL-10 production (Figure 3D).

**Figure 4:**
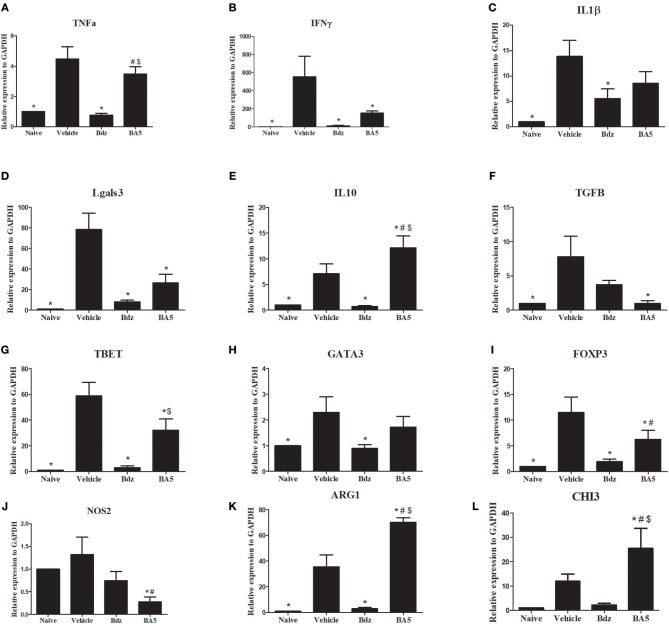
Gene expression in the hearts of infected mice after BA5 or benznidazole treatment. Analysis of gene expression was performed by real-time qRT-PCR using cDNA samples prepared from mRNA extracted from hearts of naive and chronic Chagasic mice treated with vehicle (Saline), BA5 (10 mg/Kg) or Benznidazole (Bdz; 100 mg/Kg). **(A)** *Tnfa*, **(B)** *Ifn*ɤ, **(C)** *IL1b*, **(D)** *Tgfb*, **(E)** *Il10*, **(F)** *L*g*als3*, **(G)** *FoxP3*, **(H)** *Tbet* **(I)** *Gata3* **(J)** *Nos2*, **(K)** *Arg1* and **(L)** *Chi3* gene expression. Values represent the means ± S.E.M. of 5 mice (naïve) or 10 mice (infected) per group. *P < 0.05 compared to vehicle-treated mice; ^#^*P* < 0.05 compared to naive group. ^*$*^*P* < 0.05 compared to benznidazole-treated group.

To evaluate the effects of BA5 treatment in the heart, we performed RT-qPCR analysis in samples from the different experimental groups. As expected, infection upregulated the expression of several inflammation-related genes, including TNF-α, IFN-γ, IL-1β, galectin-3, TGFβ, and IL-10, compared to naïve controls (Figures 4A–F). Treatment with BA5 (10 mg/Kg) or benznidazole promoted a significant reduction in the gene expression of IFN-γ, TGFβ and Gal-3, but not in gene expression of TNF-α, and IL-β and (Figure 4). In contrast, IL-10 was significantly increased by BA5, while treatment with benznidazole reduced IL-10 transcripts (Figure 4E). Moreover, the gene expression levels of T-bet, GATA-3, and FoxP3, transcription factors associated with T-cell subtypes T helper 1, T helper 2 and Treg cells, respectively, was investigated. These transcription factors were significantly increased in vehicle-treated mice compared to naïve controls. BA5 promoted a significant reduction in Tbet and FoxP3 gene expression, but not on GATA-3 gene expression, while benznidazole reduced the transcription of all three factors (Figures 4G–I).

Based on the critical role of macrophages in the heart inflammatory infiltrate in Chagas disease, we evaluated the gene expression of markers for M1 and M2 macrophages. Treatment with BA5, but not with benznidazole, promoted a significant reduction in the M1 marker iNOS (Figure 4J). Conversely, a marked increase in the M2 markers Arg1 and CHI3 was seen after BA5 treatment, while this marker was reduced in benznidazole group (Figures 4K,L).

### Assessment of Parasite Load in Mice Treated With BA5

**Figure 5:**
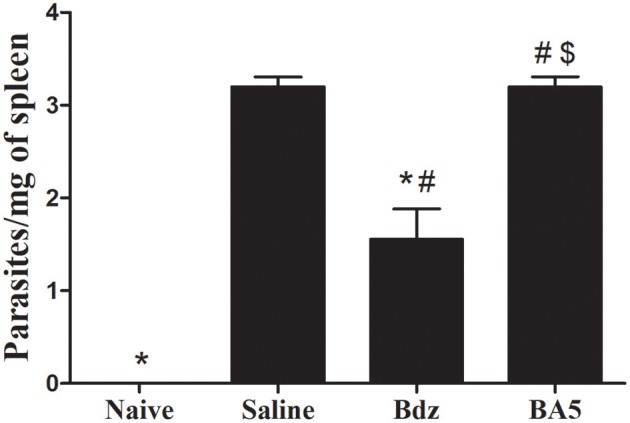
Effects of BA5 treatment in the residual parasite load. Spleen fragments obtained from uninfected and *T. cruzi*-infected mice treated with vehicle, Bdz or BA5 were used for DNA extraction and RT-qPCR analysis for quantification of parasite load, as described in the Materials and Methods section. Values represent the means ± S.E.M. of 5 mice (naïve) or 10 mice (infected) per group. *P< 0.05 compared to vehicle-treated mice; ^#^*P* < 0.05 compared to naive group. ^*$*^*P* < 0.05 compared to benznidazole-treated group.

The use of immunosuppressive drugs during chronic Chagas disease has been associated with parasitemia resurgence (21). Thus, we evaluated blood samples during the course of the treatment with BA5. All samples evaluated at different time points were negative for *T. cruzi* trypomastigotes (data not shown). In order to determine if BA5 treatment affected the residual parasite load, we performed RT-qPCR in spleen samples from infected mice submitted to the different treatments. As shown in Figure 5, treatment with BA5 at 10 mg/Kg did not influence the parasite load. In contrast, treatment with benznidazole caused a significant reduction in the parasite load when compared to vehicle-treated mice.

## Discussion

Current treatment for Chagas disease is based on trypanocidal drugs in the intention of eliminate parasitic load (22). This approach has proven to be useful in the management of acute Chagas disease, but inefficient in the majority of patients, which are in the chronic stage of the disease (10, 23). Regarding chronic Chagas disease cardiomyopathy, the main problem is the persistence of an intense inflammatory response, which leads to a permanent structural damage in myocardium and culminates with heart dysfunctions (24, 25). Therefore, the development of strategies to attenuate inflammatory response without affecting parasite control can be of great value for CCC patients. In the present study, we investigated the effects of BA5, an amide semi-synthetic derivative betulinic acid, in a mouse model of chronic Chagas cardiomyopathy which reproduces the pathological findings observed in human hearts (19). We found here a potent immunomodulatory potential of BA5, reducing inflammation and fibrosis in the hearts, as well as the expression of important inflammatory mediators which participate in the regulation of pathogenesis of CCC. Importantly, this immunomodulatory action was not accompanied by an increase in parasite load.

In a previous study we have shown that BA5 modulates the *in vitro* activation of T lymphocytes through a mechanism related to the inhibition of calcineurin (14), an enzyme that regulates the activation of the nuclear factor of activated T cells, NFAT which is involved in T cell activation (26). *In vivo*, BA5 was shown to inhibit edema formation in a delayed type hypersensitivity model (14), which is a T cell dependent reaction. The finding that BA5 inhibits chagasic myocarditis, which is an inflammatory reaction similar to DTH response, composed mainly by macrophages and lymphocytes, reinforces its modulatory role upon T cell-mediated immune responses.

Inflammation and disease severity in CCC have been associated with high levels of IFN-γ. We found here a marked reduction in IFN-γ after BA5, both in the serum as well as in the heart tissue, having thus systemic and local modulatory effects. The modulation of Th1-type responses was reinforced by the reduction of gene expression of Tbet, a Th1 associated transcription factor, in the hearts of infected mice after BA5 treatment. Similar results were found in mice treated with benznidazole.

A marked increase in IL-10 production was seen after BA5 treatment, both in the sera and hearts of chronic chagasic mice, a feature which was not achieved with benznidazole treatment. IL-10 is a crucial regulatory cytokine, and its production is associated with a better outcome of chronic Chagas disease, since asymptomatic individuals were found to produce higher IL-10 levels than patients with cardiomyopathy (27). IL-10 negatively correlated with IFN-γ levels in chronic chagasic individuals, suggesting a protective effect against the type 1 inflammatory response (28).

Macrophage activation and production of pro-inflammatory mediators, such as TNF-α, have been well demonstrated to play a critical role for parasite control during the acute phase of infection. During the chronic phase, however, these cytokines are well associated with chronic inflammation and cardiac dysfunction in Chagas disease. BA5 and its prototype betulinic acid down regulate key inflammatory mediators such as TNF-α *in vitro* and *in vivo*, regulating the activation of the transcription factor NF-κB in activated macrophages (14, 29). In the present study we confirmed the anti-TNF-α action of BA5 in chronic *T. cruzi* infection.

An interesting observation in our study was a switching in macrophage polarization from M1 to M2, evidenced by a decrease in the expression of NOS2 and proinflammatory cytokines and the increase in the M2 markers, Arg1 and CHI3, in the hearts of mice treated with BA5. This effect was not achieved by treatment with benznidazole. M2 macrophages play a protective role in several disease settings, including cardiovascular diseases, due to their anti-inflammatory properties (30). IL-10 production is elevated in M2 macrophages, and greatly contributes to their anti-inflammatory action. Thus, it is likely that the increase in IL-10 production seen after BA5 treatment is in part due to the M2 polarization.

The balance between the parasite-mediated immune response and the inflammation detrimental to host tissues probably determines the course of CCC (9). We have previously shown that BA5 presents anti-*T. cruzi* activity (13). In the present study, however, by quantifying the *T. cruzi* DNA in the spleen we found that treatment with BA5 did not affect parasite load. The lack of parasite reduction by BA5 may be explained by a low efficacy of parasite clearance during the chronic phase, or alternatively, the antiparasitic effects of BA5 may be surpassed by its immunosuppressive effects. Moreover, our data reinforce the hypothesis that heart inflammation does not correlate directly with parasite load (31).

In agreement with a previous study (32), we observed a reduction in parasite load in benznidazole-treated chronic chagasic mice, accompanied by a reduction of cardiac inflammation, fibrosis and crucial inflammatory mediators such as TNF-α and IFN-γ. Several studies have shown immunomodulatory effects of benznidazole. More specifically, benznidazole inhibits the production of inflammatory mediators, including IL-6, NO and TNF-α (33–35). In addition, in a cecal ligation and puncture mouse model, it has been demonstrated that benznidazole treatment reduces mortality by down-regulating NF-κB and mitogen-activated protein kinase (MAPK) (p38 and extracellular signal-regulated kinase - ERK) (34). In fact, benzonidazole acts as an imunomodulador agent, suggesting that the beneficial properties of benzonidazole are related on both trypanocidal action and immunomodulatory effects (36, 37).

Despite the reduction of inflammation and fibrosis, we did not observe any significant gains in cardiac function by EKG and ergometry analyses, both in BA5 or benznidazole treated mice. Since *in vitro* drug combination showed a synergistic effect (13), it is possible that a combined therapy may result in a better recovery, especially if given earlier.

Finally, we conclude that the betulinic acid derivative BA5 had a potent anti-inflammatory activity on a model of parasite-driven heart disease, being related to elevated IL-10 production and a switch from M1 to M2 macrophage subset. More importantly, decrease in inflammation and fibrosis was achieved without affect parasite control, making BA5 an interesting molecule for development of alternative treatments for patients with CCC.

## Data Availability

The raw data supporting the conclusions of this manuscript will be made available by the authors, without undue reservation, to any qualified researcher.

## Ethics Statement

All experiments were carried out in accordance with the recommendations of Ethical Issues Guidelines, and were approved by the local ethics committee for animal use under number 001/15 (FIOCRUZ, Bahia, Brazil).

## Author Contributions

CM, ES, RS, JV, IO, CN, BB, AC, and DS performed the experiments. CM, CN, SM, JB-F, DM, and MS anaylzed the data. CM, JB-F, DM, and MS conceived the study and wrote the manuscript.

### Conflict of Interest Statement

The authors declare that the research was conducted in the absence of any commercial or financial relationships that could be construed as a potential conflict of interest.
